# Distributed processing of color and form in the visual cortex

**DOI:** 10.3389/fpsyg.2014.00932

**Published:** 2014-10-27

**Authors:** Ilias Rentzeperis, Andrey R. Nikolaev, Daniel C. Kiper, Cees van Leeuwen

**Affiliations:** ^1^Institute of Neuroinformatics, University of Zürich and Swiss Federal Institute of TechnologyZürich, Switzerland; ^2^Laboratory for Human Systems Neuroscience, RIKEN Brain Science InstituteWako, Japan; ^3^Laboratory for Perceptual Dynamics, University of LeuvenLeuven, Belgium

**Keywords:** color, form, segregation, integration, distributed processing, mixed selective cells, high dimensional code, complex selectivity

## Abstract

To what extent does the visual system process color and form separately? Proponents of the segregation view claim that distinct regions of the cortex are dedicated to each of these two dimensions separately. However, evidence is accumulating that color and form processing may, at least to some extent, be intertwined in the brain. In this perspective, we review psychophysical and neurophysiological studies on color and form perception and evaluate their results in light of recent developments in population coding.

## INTRODUCTION

The seminal investigations of Hubel and Wiesel () established that the receptive field properties of single neurons in V1 emerge from the integration of neurons in the previous processing stage. Since then, it is commonly believed that visual information is processed in a hierarchical fashion, consisting predominantly of feedforward feature integration and convergence. Distinct streams of processing are initially kept anatomically separate, only to come together in higher order areas by specialized cells (Barlow, 1972) – or, alternatively, to be organized functionally, in time through synchronized activity (Von Der Malsburg, 1994; Singer and Gray, 1995).

In this perspective, the earlier stimulus representations are characterized by segregation. The segregation hypothesis postulates that different attributes of visual stimulation are being received and processed by distinct populations of neurons. (Zeki, 1978; Hubel and Livingstone, 1987; Livingstone and Hubel, 1988) and contrasts with integrality, which permits populations of neurons to have mixed selectivities.

More recent studies have taken issue with the notion of segregation (see Lennie, 1998; Gegenfurtner and Kiper, 2003; Shapley and Hawken, 2011 for reviews) as the most viable option for understanding visual representation. It has been shown, for instance, that motion and disparity are encoded jointly in certain subpopulations of neurons (Roy et al., 1992; DeAngelis et al., 1998; Anzai et al., 2001; Pack et al., 2003; Grunewald and Skoumbourdis, 2004). Such results suggest that it may be time to reconsider the segregation model.

Here we will review the status of the evidence for segregation of color and form. This is of special interest because color and form are most likely processed along the ventral cortical pathway (Ungerleider and Mishkin, 1983) and yet, this pair has been regarded as extremely segregated, not only represented in separate neurons, but also in distinct brain regions. For this reason, segregation of color and form is more controversial than that of color or form versus motion. Dubner and Zeki (1971) first observed a region in the superior temporal sulcus of the macaque that was selective for direction of motion but unresponsive to color or orientation. Zeki (1973, 1974, 1977) showed evidence that areas in extrastriate cortex functionally differ from each other; he proposed that area V4 is specialized for color and V5 (MT) for motion. In later studies, Hubel and Livingstone (1987; Livingstone and Hubel, 1988) observed that color and form are processed in distinct regions within both the primary (V1) and prestriate (V2) cortex. Motion is considered to be processed along the dorsal stream. Lennie (1998) argued that motion could be a special case and “the separation of motion signals from signals about other dimensions of image variation means the analysis they subserve is self-contained.” We will not contest this here.

We concentrate on the ventral stream, and investigate if any further subdivisions are necessary. We do not wish to claim, however, that visual object information is exclusively processed in the ventral stream. There is evidence that object representations exist in parallel in both dorsal and ventral streams (Konen and Kastner, 2008). The dorsal information pathway is thought to be involved in the encoding of spatial relationship of objects (Mishkin et al., 1983). It could thus be the case that dimensions related to spatial relationships among objects are anatomically separate from the ones that define an object.

In our present review of color and form segregation we argue, first, that the segregation view does not square well with behavioral findings, including those on attentional feature integration that have traditionally been interpreted in a segregation-friendly framework (Treisman and Gelade, 1980). Next, we re-examine some of the classical neurophysiological studies demonstrating segregated processing of color and form, along with more recent evidence that may undermine the necessity of segregation as the best possible explanation. In areas predominantly representing color or form, weak selectivities for the other feature also exist. The predominance of studies in single neurons has hitherto obscured the role of weak selectivities in distributed coding. Weak selectivities can have a strong collective effect in a neuronal population. Their presence, and generally that of mixed selectivities in single neurons, enables neuronal population codes with flexible and context sensitive feature representations, properties that have been shown to exist in early visual cortex. We discuss how neuronal population codes could be used in perceptual integration of color and form via feedforward, and feedback and horizontal (recurrent) perceptual mechanisms.

## BEHAVIORAL STUDIES

### FORM SENSITIVITY TO COLOR AND LUMINANCE SIGNALS

According to the segregation framework, functions dedicated to color vision will be poor at form processing and those engaged in form vision operate almost exclusively on luminance signals. However, psychophysical studies have rallied against such a dichotomy by showing comparable orientation discrimination thresholds for color and luminance stimuli (Webster et al., 1990; Reisbeck and Gegenfurtner, 1998; Beaudot and Mullen, 2005). Likewise, performance in contour integration was similar from either color or luminance local elements (McIlhagga and Mullen, 1996; Rentzeperis and Kiper, 2010). Furthermore, recent rating experiments on the similarity of two bars varying in both orientation and color have been inconclusive on how separable color and orientation are (Bimler et al., 2013). This evidence suggests the possibility of color and orientation mechanisms interacting at an early stage of visual processing.

Two well-known visual illusions in the perception of orientation are the tilt aftereffect and the tilt illusion (Gibson and Radner, 1937). Livingstone and Hubel (1987b) reported that there is no tilt illusion effect at isoluminance, a finding that supported their proposition that color and form are processed separately. However, a later study showed the presence of large tilt illusions for isoluminant stimuli (Clifford et al., 2003b). Furthermore, Flanagan et al. (1990) showed that the tilt aftereffect can be also induced by isoluminant gratings. In showing that color-only channels are sensitive to the illusion, both studies (Flanagan et al., 1990; Clifford et al., 2003b) support the interaction of color and form early in processing.

Contrast sensitivity as a function of spatial frequency for both red-green and blue-yellow isoluminant gratings initially was shown to be low pass (Mullen, 1985), a finding that supported the view that color vision has poor spatial acuity (Livingstone and Hubel, 1988). However, later studies pointed out that the low pass contrast sensitivity function is the envelope of several band-pass spatial frequency filters (Bradley et al., 1988; Losada and Mullen, 1994).Mechanisms that have band-pass filters are suitable for the detection of edges or locally oriented elements that form global patterns. These results, therefore, indicate that color vision, like luminance vision, encodes the visual scene using band-pass filters.

### COLOR SELECTIVITY OF LOCAL AND GLOBAL FORM PROCESSES**:** GLASS PATTERN STIMULI

Psychophysical studies of (achromatic) form processing mechanisms have often used Glass patterns as stimuli (Glass, 1969; Glass and Switkes, 1976; De Valois and Switkes, 1980; Kovacs and Julesz, 1992; Dakin, 1997; Wilson et al., 1997; Wilson and Wilkinson, 1998; Dakin and Bex, 2001, 2002). Glass patterns are constructed from oriented dot pairs; depending on the orientation of the dot pairs different global forms can be perceived. Wilson and Wilkinson (1998) proposed a feedforward, hierarchical model of Glass pattern processing, in which the early stages (V1/V2) use oriented filters and rectification to process the local dot pairs and later stages (V4) pool and sum the output of previous stages to create the percept of global form. Accordingly, subsequent electrophysiological studies have indicated that V1 and V2 neurons respond to dot pairs irrespectively of global form (Smith et al., 2002, 2007).

Colored Glass patterns are an eminent tool for studying color selectivity of local and global form processes. Mandelli and Kiper (2005) measured detection thresholds for circular Glass patterns that consisted of dots isoluminant to the background, with different colors within each dot pair. When the difference in color between dot pairs increased, observer sensitivity decreased. The results suggest that there are local processing mechanisms with narrow color tuning (color selectivity) that are also orientation selective. If not we would expect the observer sensitivity to stay the same irrespective of the presence of a color difference between dot pairs. The average tuning in color space of the local mechanism (the range of colors a local unit responds to) was consistent with the physiological observations that color selective cells in V1 and V2 are also orientation selective (Leventhal et al., 1995; Friedman et al., 2003). The result, therefore, is in accordance with the notion that early processing mechanisms show mixed selectivity.

To probe the color selectivity of global form mechanisms, Wilson and Switkes (2005) measured Glass pattern detection when the colors between dot pairs [but not within dot pairs as in the Mandelli and Kiper (2005) study] were varied. They found that the distance in color between the dot pairs did not affect observer sensitivity. This result suggests a color invariant global form mechanism. Adaptation studies with color and luminance Glass patterns confirmed this result and showed that global form mechanisms are invariant to luminance polarity as well (Rentzeperis and Kiper, 2010; Rentzeperis et al., 2012). In summary, the results on colored Glass patterns indicate that early form processes that code for local features are also selective for color; however, intermediate processes that pool and sum the local orientation cues are color invariant, in the sense that they can integrate oriented signals of any chromaticity.

### COLOR AND FORM ASYNCHRONY

In a series of psychophysical studies, Moutoussis and Zeki (1997a,b) showed that different visual features presented at the same time may not be perceived as simultaneous. That these features are perceived at different times, the authors argued, indicates that they are processed separately. In one of these studies, participants were shown on one half of the screen a colored checkerboard pattern (the colored squares alternating from red to green) and on the other half grey bars (all alternating their tilt from left to right). Participants had to match the colors of the squares with the orientation of the bars that were presented at the same time (Moutoussis and Zeki, 1997b). Both color and orientation changes occurred at the same rate but their phase difference varied. For certain phase differences the color and orientation pairs perceived were different from the actual ones. The temporal mismatch indicated that color is perceived approximately 63 ms before orientation. Bartels and Zeki (1998) argued that this kind of perceptual asynchrony supports functionally distinct modules in the brain which are acting as autonomous perceptual units, each processing the stimulus in their own time frame (Bartels and Zeki, 1998; Zeki and Bartels, 1998). This claim, however, is at odds with electrophysiological measurements on the macaque, which have shown that the difference in visual response latencies between visual areas does not exceed 20 ms (Schmolesky et al., 1998). If different visual areas in the brain acted as independent functional and perceptual units we would expect the latency in neural response between different visual areas to match the time difference between color and orientation perception.

Holcombe and Cavanagh (2001) measured the temporal resolution of the perception of feature pairs when color and orientation were spatially separated and spatially superimposed. In both conditions, color and orientation changes happened at the same time and participants had to match them. When color and orientation were spatially separated participants reached 75% threshold accuracy in reporting the correct pairings for rates of presentations that were less than 3 Hz. However, when color and orientation were spatially superimposed participants reached the same performance for rates of presentations that were more than six times faster. The latter frequency corresponds to ∼50 ms for feature binding. The authors concluded that color and form are processed in combination in early stages; when the two features are spatially separated they go through a binding process which has low temporal resolution.

In a subsequent study, Clifford et al. (2003a) used sinusoidal gratings oscillating in color and orientation at the same temporal frequency and for a range of phase differences. They found that for rapid presentation rates (10 Hz) both color and orientation were perceived at the same time. However, as the presentation rates decreased the asynchrony between color and orientation grew; for a presentation rate of 1 Hz, color perception preceded orientation perception by 50 ms. The authors proposed that the perceptual asynchrony observed for slow presentation rates could be attributed to a difference in adaptation between color and form processes, resulting in changes to their temporal response profiles. They suggested, however, that both color and orientation are processed by overlapping populations of neurons (since participants show high temporal precision) with each neuron in this population using multiplexed temporal codes for color and orientation. This interpretation is in line with electrophysiological measurements in monkeys indicating that separate temporal codes representing color and form are multiplexed in single neurons in areas V1, V2, and V4 (McClurkin and Optican, 1996; McClurkin et al., 1996). The data from these studies were in accordance with a model in which the response of a neuron to a colored form is the product of a response pattern encoding color and a response pattern encoding form added on top of the neuron’s average response to all stimuli (McClurkin et al., 1996).

### FEATURE INTEGRATION THEORY AND VISUAL SEARCH

The psychophysical literature suggests early integration of color and form information. This calls into question theories proposing that visual feature integration takes place in a late stage of processing. Among these theories, feature integration theory (FIT; Treisman and Gelade, 1980), has been the most influential. FIT claims that color and orientation are initially processed in parallel and pre attentively. As a result, the detection time of a red target remains approximately constant irrespective of the number of green distracters in the visual scene. Note that the target has a basic feature that is not shared by the distracters. By contrast, the detection time of a horizontal red target amongst horizontal green or vertical red distracters increases as the number of distracters grows. Here, the target shares a basic feature with each of the distracters so only their combination is distinctive. Searching targets based on integral, combined, features is done serially, on an item-by-item basis (Treisman and Gelade, 1980). Perceptual integration, therefore, involves attention.

In line with FIT, several authors have proposed biologically plausible models of visual search in which visual stimuli are processed in parallel by feature maps, each covering the entire visual field and representing a single basic visual feature. Feature maps identify locations in the visual field where the feature they represent is different from its surrounding. All the feature maps then feed into a saliency map which codes for conspicuous locations irrespective of the visual feature that stands out (Koch and Ullman, 1985; Wolfe, 1994; Itti et al., 1998). The existence of a feature map for each feature does not necessarily imply an independent physiological locus for that map (Wolfe, 1994).

The locus of the saliency map is not clear; based on neurophysiological or imaging data several candidates regions have been proposed in the parietal cortex (Gottlieb et al., 1998; Geng and Mangun, 2009), V4 (Mazer and Gallant, 2003), FEF (Thompson and Bichot, 2005; Serences and Yantis, 2007), and superior colliculus (Kustov and Robinson, 1996). Li (1999, 2002) has proposed that V1 acts as a saliency map and that no separate layer of feature maps is needed; the receptive fields of the neurons that have the highest responses (regardless of the neurons’ feature selectivity) indicate the salient location(s). Recent physiological evidence in humans is consistent with this observation (Zhang et al., 2012).

A number of results, however, have contested the interpretations of FIT and related computational models. For instance, visual search for targets defined by a conjunction of motion and form features (McLeod et al., 1988) and for 3D shapes (Enns and Rensink, 1990) happens in parallel. Visual search for targets and distracters oriented differently can be serial for certain orientation combinations (Wolfe, 1994), even though neurons encode orientation in early visual areas. Finally, visual search that initially was serial for certain stimuli can become parallel with practice (Sireteanu and Rettenbach, 1995).

Why, if color and form are not segregated, does search for a unique feature appear parallel, while search for a conjunction of color and form appear serial? Recently, Rosenholtz et al. (2012) proposed a model that aims to explain these results. The model assumes that the visual system computes a set of summary statistics pooled over local regions that cover the whole visual field. The local regions grow linearly with eccentricity so as to represent the degraded resolution of the visual system for peripheral locations attributed to the larger receptive fields in the periphery compared to the fovea. During a search task, the visual system has to discriminate the summary statistics of peripheral regions with distracters only from those containing the target. If peripheral vision can discriminate the target from the distracters, visual search will be parallel, because the subjects will have information that will guide their eyes to the target right away. If peripheral vision cannot discriminate the target from the distracters, visual search has to be serial because subjects will not have information on where to move their eyes to track the target. In the context of this model, feature binding is largely independent of top-down attention; search performance depends on the amount of information loss of the visual system mainly in the periphery. Thus, the model could operate with either segregated or integrated processing of features in early visual cortex. Rosenholtz (2011) suggested that summary statistics may be computed in multiple color bands, possibly including correlations across bands. Computing summary statistics within a color band means computing responses of orientation-selective, band-pass filters within a color band, reminiscent of filters that are both orientation and color selective.

In sum, psychophysical evidence supported an integrated rather than a segregated view on color and form processing. Model studies show the viability of such integrated views, or are at least agnostic with respect to the controversy. In the following section we consider the classical neurophysiological studies in support of segregation of color and form in early visual cortex and contrast them with more recent findings that show significant intermixing of color and form in the same areas.

## SEGREGATED OR INTEGRATED SELECTIVITIES OF SINGLE NEURONS IN EARLY VISUAL CORTEX?

Early, influential studies on cortical processing have shown evidence of spatially separate populations of neurons being sensitive to different features of a visual scene. For one, studies in V1 and V2 of the primate cortex have indicated regions with distinct anatomical characteristics. Staining with mitochondrial enzyme cytochrome oxidase (CO), revealed alternating dark and light regions in layers 2/3 of V1, a result that indicates high and low concentrations of CO respectively in V1 (Humphrey and Hendrickson, 1980; Horton and Hubel, 1981). The darker stains in sections tangential to the cortical surface were coined *blobs*, in accordance with their three dimensional, oval shapes, and the lighter stained regions were called *interblobs*. V2 shows a different, but equally interesting pattern of patches when stained for CO. Instead of oval shapes, tangential sections show an alternation of dark stripe and light interstripe regions; the dark stripes are of two types; thick and thin ones (Livingstone and Hubel, 1982; Tootell et al., 1983). From tracer injections, Livingstone and Hubel (1984, 1987a) showed evidence that the thin stripes are connected to the *blobs*, the interstripes to the *interblobs*, and the thick stripes to layer 4B of V1.

**FIGURE 1:**
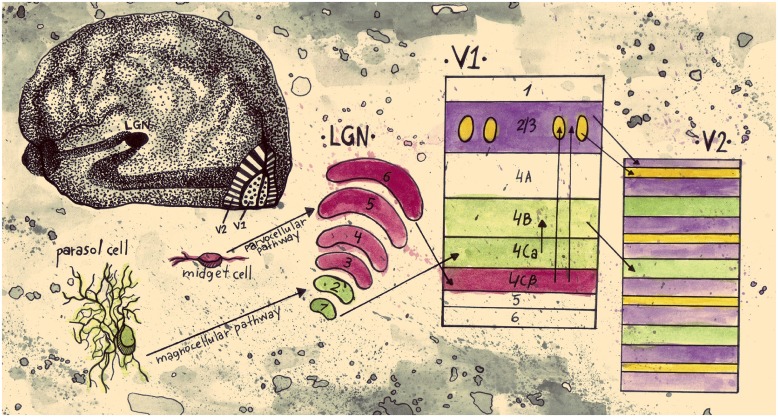
**Schematic representation of an early segregation model of visual information pathways from the retina to V2.** Parasol cells in the retina are linked to the magnocellular pathway. They project to layers 1 and 2 of LGN, continue to layer 4Cα of V1, and then from layer 4B of V1 they project to the thick stripes of V2. This pathway conveys information about motion and stereo. Midget cells in the retina are part of the parvocellular pathway; they project to layers 3–6 of LGN and on to layer 4Cβ of V1. From then on they split into two streams. The stream that conveys information about color projects to the blobs in layers 2/3 of V1 and then to the thin stripes in V2. The stream that conveys information about form projects to the interblob area in layers 2/3 of V1, and then to the interstripes in V2 (drawn by Anastasia Lavdaniti; anastasialavdaniti@gmail.com).

Livingstone and Hubel (1984, 1988) proposed that the anatomically segregated regions in early visual cortex have distinct functional properties. They suggested a link between the CO regions and the magnocellular (M) and parvocellular (P) retino-geniculo-cortical pathways. Whereas the M pathway projects from layer 4B in V1 to the thick stripes in V2 and is selective for depth and motion the P pathway is subdivided into two streams; one passing through the *blobs* in V1 and the thin stripes in V2 that mediates color and another one passing through the *interblobs* in V1 and the interstripes in V2 that mediates form (**Figure 1**). The authors concluded that double opponent cells (cells exhibiting both color and spatial opponency) in V1 *blobs* are not orientation selective and have low spatial acuity. Edges, they suggested, are signaled by cells in the *interblob* area in V1. While these cells are orientation selective, they are not color opponent; they can respond to a luminance or color edge regardless of its color but cannot code the color information of the edge (Hubel and Livingstone, 1987; Livingstone and Hubel, 1988). Additional physiological studies have supported the idea that within area V2, separate anatomical regions have distinct functional properties (Hubel and Livingstone, 1985, 1987; Shipp and Zeki, 1985; Tootell and Hamilton, 1989; Ts’o et al., 1990; Malach et al., 1994; Roe and Ts’o, 1995; Moutoussis and Zeki, 2002).

Since then a number of electrophysiological studies have challenged this segregated view on V1 and V2. Lennie et al. (1990) measured the responses of cells in layers 2/3 of V1 and found that cells inside and outside *blobs* did not have different chromatic properties. Friedman et al. (2003) measured, in layers 2/3 of V1 and in V2, the selectivity of cells for color, orientation and border position from alert macaque monkeys. They found no correlation between any of the selectivities. Leventhal et al. (1995) recorded cells from layers 2/3 and 4 in V1 and also found no correlation between orientation and color selectivity. Clearly, based on the segregation view, a negative correlation would have been predicted. Similarly to Lennie et al. (1990), there was no difference observed in the response properties between cells outside and inside the V1 *blobs* (Leventhal et al., 1995). Using implanted 100 electrode arrays in V1, Economides et al. (2011) found very subtle differences in orientation tuning between neurons in *blobs* and *interblobs*; the mean orientation bandwidth of cells in *blobs* was 28.4 and in *interblobs* 25.8°. The most pronounced difference was in activity: *blob* cells had 49% higher firing rates than *interblob* cells.

A CO *blob* system has also been found in primates with no color vision (Condo and Casagrande, 1990). O’Keefe et al. (1998) measured the response of V1 neurons in the nocturnal, New World monkey (a species containing only a single cone type). They found no difference in orientation tuning, eye dominance, temporal frequency tuning and contrast response for neurons in *blobs* and *interblobs*. The repeating anatomical patterns found in the visual cortex and other parts of the brain, Purves et al. (1992) argued, do not reflect a fundamental functional principle but rather are byproducts of developmental requirements.

Johnson et al. (2001) divided the neurons from which they recorded in V1 into three groups, depending on their sensitivity to color and spatial patterns of luminance. Most of the neurons strongly preferred luminance patterns compared to colored ones (60% luminance cells); fewer neurons showed strong color selectivity (11% color cells). Interestingly enough, a considerable percentage of neurons were selective to both luminance and color patterns (29% color-luminance cells). Color-luminance cells did not respond or responded poorly to patterns of low spatial frequency (<0.5 cycles per degree); instead they showed a band-pass tuning similar to luminance cells. Most color cells were low pass in their spatial frequency tuning. In a later study Johnson et al. (2004) concluded that color-luminance cells are double opponent. In contrast to the Hubel and Livingstone studies, Johnson et al. (2008) found that most double opponent color-luminance cells are also orientation selective.

**FIGURE 2:**
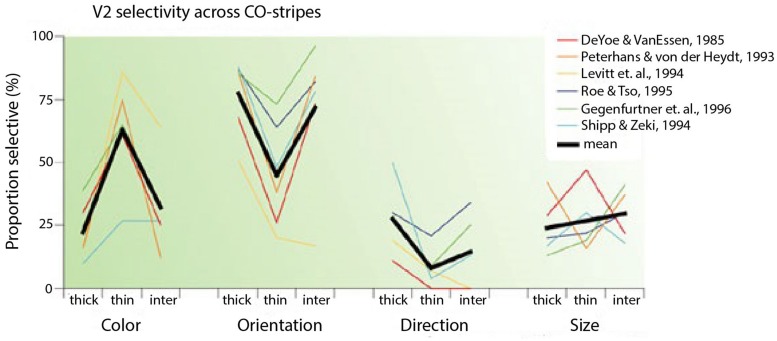
**Selectivity of V2 neurons in different CO compartments (taken from Gegenfurtner, 2003).** The graph shows selectivities of cells for color, orientation, direction and size in thick, thin and inter-stripes in V2 from six different studies. The black lines are the average selectivities from the six studies.

In reviewing the functional segregation of early visual areas, Gegenfurtner (2003) collected results from six studies in which cells from the distinct CO compartments in V2 (thin stripes, thick stripes, interstripes) were examined (DeYoe and Van Essen, 1985; Peterhans and von der Heydt, 1993; Levitt et al., 1994; Roe and Ts’o, 1995; Gegenfurtner et al., 1996; Kiper et al., 1997). According to the segregation perspective, cells in the interstripes are selective to form and cells in the thin stripes are selective to color. The averages from these studies confirmed that cells in the thin stripes are most selective for color, cells in the thick stripes and interstripes are most selective for orientation and cells in the thick stripes are most selective for direction of movement (**Figure 2**). Nevertheless, cells in each compartment were selective for other features as well. The results show that to a considerable extent, the selectivities within both the interstripe and thin stripe regions are mixed, especially for color and form. Around 30% of cells in the interstripes are selective for color and around 40% of cells in the thin stripes are selective for form.

Several studies discussed in this section have questioned the hypothesis that neurons in different CO compartments process separate dimensions of the visual scene. If, on the one hand, there is some degree of anatomical and functional specialization in the brain, why are there these mixed selectivities in the different CO compartments? Or why are there neurons with more than one selectivity in the early visual cortex? If, on the other hand there is no anatomical and functional segregation in the brain why is there a bias for certain features in different CO compartments?

All of the above-mentioned electrophysiological studies analyzed neural activity as if each neuron acted as an independent computational unit, i.e., without considering the possible role of interactions between neurons. Individual neurons with broad selectivities to color or to orientation were categorized as non-selective to color or to form, respectively. Yet, perhaps, perceptually significant information does not arise at the single neuron level, but from a population of neurons. The combination of responses from a population of neurons may reveal robust decoding for conditions where individual neurons show broad selectivity. Neurons, as we discuss in this section, could have mixed selectivities with unequal tuning widths for different features; however, a population code consisting of inputs from neurons like that may show sharp tuning for all features. In the next section we discuss studies that examine possible ways a population of neurons can encode information and what are the attributes of neurons that make encoding of information optimal.

## DISTRIBUTED PROCESSING

Early influential studies on color and form processing promoted the view that perceptually significant information happens at the single neuron level (Zeki, 1978; Livingstone and Hubel, 1988). An analysis adhering to this view can overlook the possibility that weak selectivities at the single neuron level encode information at the population level. Evidence supports the notion that the brain processes information by combining signals from neuronal populations. Firstly, repeated presentations of the same stimulus evoke considerably variable responses from a single neuron (Tolhurst et al., 1983; Vogels and Orban, 1990). If the activity of single neurons represented perceptually significant activity we would expect less variability in the response of a neuron after repeated presentations of the same stimulus. This leads us to the next point; single neurons in the visual cortex have weak correlations with behavioral decisions (Britten et al., 1996; Shadlen et al., 1996). Finally, the structural features of neurons suggest the formation of distributed circuits with long range connectivity (Alexander and van Leeuwen, 2010; Yuste, 2011).

Contrasting with the single neuron viewpoint, the response of a single neuron gives an ambiguous response by itself and can only provide sufficient information if considered in conjunction with the responses of the rest of the neurons forming a network. In line with this perspective, Lehky and Sejnowski (1988) showed that selectivity of single neural units could give misleading information on the function of a neural network. Population coding analysis examines how information is represented from the pattern activity in a group of neurons. In an influential study on population coding, Georgopoulos et al. (1986) represented the activity of each neuron recorded in the arm area of the primate motor cortex as a vector pointing in a specific direction in 3-D space. The vector associated with each cell was weighted according to the activity of that cell, and then all the vectors were summed. The direction of the vector sum was in close approximation to the direction of the arm movement of the monkey despite the broad tuning of single cells.

**FIGURE 3:**
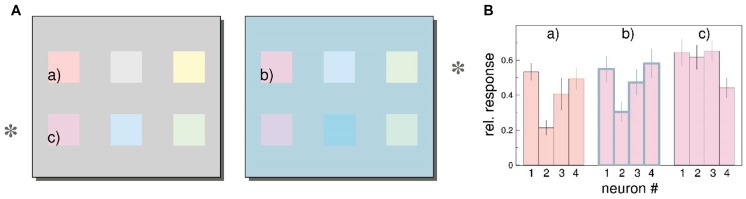
**Color induction in primary visual cortex (taken from Wachtler et al., 2003). (A)** The color patches on the rows marked by an asterisk are physically identical, but are shown on different backgrounds; thus they appear different. For example color patch (b) looks more similar to physically different color patch (a) compared to physically identical color patch (c). **(B)** Estimated responses of four neurons to patches (a)–(c). (see “Estimating Stimulus Color from Population Responses” in the Results section of Wachtler et al. (2003) for a detailed description of the analysis). The responses are normalized relative to the maximum firing rate for each neuron. The pattern of responses of the four neurons for patch (b) are more similar with the responses for patch (a) than for patch (c), even though patches (b) and (c) are physically identical. Similar results were found for a population of 94 neurons.

Wachtler et al. (2003) examined whether the activity of a population of neurons in macaque V1 can represent color perception. Population responses were expressed as vectors, with each element of the vectors representing the activity of a single neuron. The authors found that distributed neural response changes with different backgrounds corresponded with induction effects in color perception (shown in a follow-up experiment with human participants). An example of the authors’ analysis is shown in **Figure 3**. In **Figure 3A**, color patches (c) and (b) are physically identical but appear different because they are displayed on different backgrounds. Furthermore, color patches (a) and (b) appear similar even though they are physically different. In **Figure 3B**, the pattern of responses of four neurons of patch (b) is more similar to that for patch (a) than for patch (c), indicating that the population response of neurons in V1 correlates with color perception. Note that decoding in this study is represented by a vector with the activities of all the neurons. In the Georgopoulos et al. (1986) study each neuron was represented by a position vector pointing at the preferred direction of that neuron; the decoded direction was given as the weighted average of all the vectors. Thus, perhaps, the rules for information processing from a population of neurons depend on the nature of the target feature.

Our understanding of how a population of neurons could represent information has been facilitated by studies that link machine learning principles with neural processing (Buonomano and Maass, 2009; Rigotti et al., 2013). In this framework, neurons that have small responses to a particular feature or to a combination of features can be crucial in the encoding of distributed information, whereas these weak selectivities would be hard to interpret in single neuron analysis. To date, this issue has predominantly been investigated in the prefrontal cortex. In the remainder of this section we focus on neurons in prefrontal cortex. We argue in the next section that if mixed selectivity is a property of neurons throughout the cortex, a simple assumption of non-linearity will enable us to explain the often conflicting results on the selectivity of neurons in early visual cortex discussed in the previous section.

**FIGURE 4:**
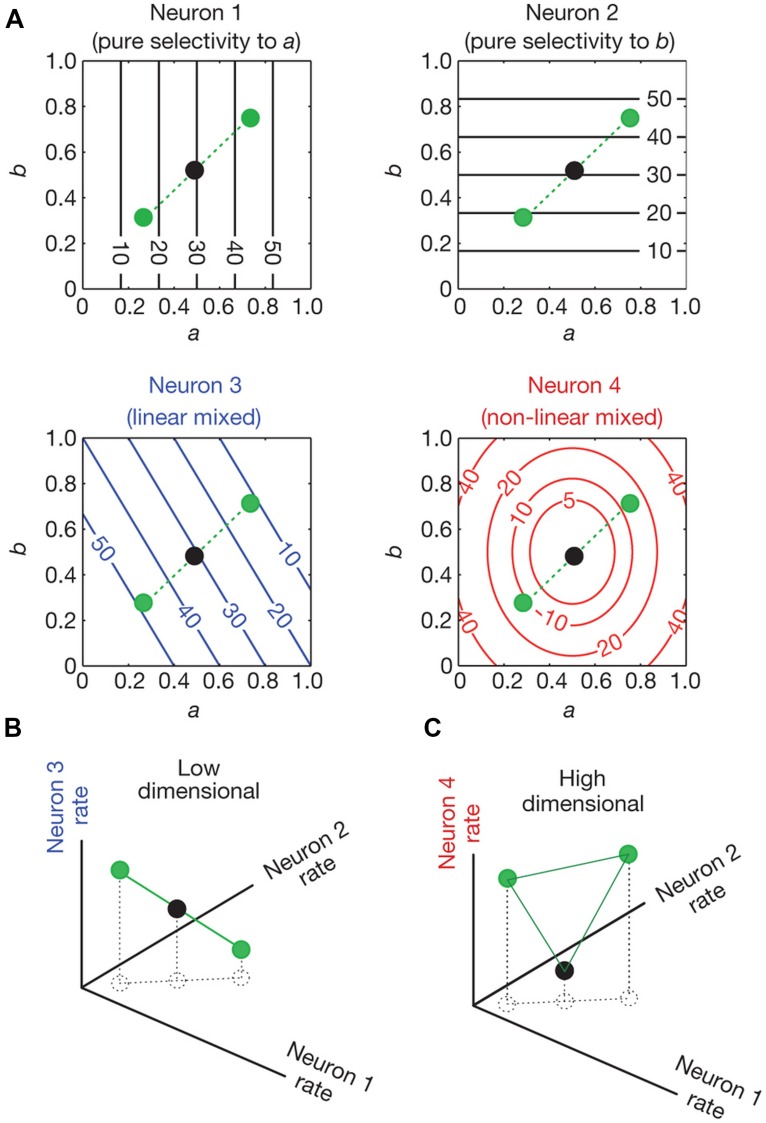
**Dimensionality of neural representations (taken from Rigotti et al., 2013). (A)** Contour plots of the firing rate of four neurons (spikes/sec). Their firing rate is shown as a function of conditions a and b which vary from 0–1. Neurons 1 and 2 are pure selective: they respond only to condition a and b, respectively. Neuron 3 is linearly mixed selective: its response is a linear combination of its firing rate to single parameters. Neuron 4 is non-linearly mixed: its response cannot be expressed as a linear combination of its firing rate to single parameters. The circles indicate the responses of the neurons for three different combinations of a and b. **(B)** The space of activities of the pure and linearly mixed neurons. **(C)**, as in **(B)**, with the only difference being that the axis where the linearly mixed neuron’s response was represented is replaced by the axis that represents the response of the non-linearly mixed neuron. The circles represent the response of the neurons for the same combinations of conditions a and b as in **(A)**. In **(B)** we see that the response of the neurons lie in low dimensional space (a line). This low dimensional space limits the possible input output relationships that a linear classifier can implement. For example a linear decoder (a two dimensional plane in this case) cannot separate the black dot from the green dots. In **(C)** where the activity of the non-linearly mixed neuron is represented, a plane not only can separate the black dot from the green dots, but it can also separate any possible combination of the three dots. This is because the activity of the neurons lies in a higher dimensional space (a plane).

In a recent study, Rigotti et al. (2013) analyzed activity of neural populations in prefrontal cortex (PFC) while monkeys performed a memory task. The authors showed that the dimensionality of the population code is higher when single neurons are tuned to a non-linear mixture of conditions compared to when they respond exclusively to one condition or a linear mixture of conditions. The concept of dimensionality generally refers to the minimum number of coordinates that are needed to fully specify all the points of a set of vectors. For example, two vectors that are linearly dependent (one is a multiple of the other) are one-dimensional (they lie on a line); if they are linearly independent they are two-dimensional (they lie in a plane). Higher dimensionality leads to a more versatile code since the number of possible classifications of a linear classifier between two conditions grows exponentially with dimensionality. This means that a population of neurons that represent information in a high dimensional space has the capacity to perform complex tasks. **Figure 4** shows neurons with different selectivities and their effect in the dimensionality of a neural population code. Neurons 1 and 2 show pure selectivity to feature a and b of some stimuli, respectively, neuron 3 shows linearly mixed selectivity to both features and neuron 4 is non-linearly selective to both features (**Figure 4A**). In **Figure 4B**, the representation of the stimuli by the pure and linearly mixed neurons is low dimensional (it is on a line). However, in **Figure 4C** we see that if we substitute one of the neurons with a non-linearly mixed one, then the representation will be on a higher dimensional space (on a plane).

Can we lose information about the selectivity of a neural population by averaging its responses? To test this hypothesis, Rigotti et al. (2013) removed the classical selectivity from a population of neurons from a set of conditions and then tested whether the conditions could still be predicted from the response of the neurons. Classical selectivity refers to the average differences between conditions. To remove classical selectivity the authors added noise that eventually makes the average responses between conditions equal. The population responses could still predict at an above chance level the condition. Thus, on the one hand, the average responses of a group of neurons showed no significant differences between conditions; on the other hand, population coding could successfully differentiate between them. This result indicates that comparison of the average responses between conditions is not sufficient for the characterization of neuron responses and that these neurons have non-linearly mixed selectivities.

As we discuss in the following section, neurons as early as in V1 show complex selectivity and thus use neural code that is of higher dimension than initially thought. Therefore, it is plausible that these neurons are non-linearly mixed. Simply averaging a population of neurons can then hide some of their selectivities. This could explain the studies with conflicting results discussed in the previous section.

From perceptron theory, it is well-known what is required for non-linear combination of selectivities. A single layer neural network can only solve linearly separable problems, and thus map similar inputs to similar outputs. Such networks cannot solve for instance the exclusive OR (XOR) problem. This problem can only be solved with the addition of a layer in the network. Barak et al. (2013) showed in a theoretical paper that starting from the extreme case of totally segregated (or linearly mixed) representations, the dimensionality of the code can increase with an intermediate layer of randomly connected neurons. Thus even if pre-cortical neurons code for single features, it is feasible for neurons as early as in V1 to have non-linearly mixed selectivities, from connections either within V1 or from higher cortical areas.

Can a neural population represent separated signals if these signals are intermixed at the single neuron level? Mante et al. (2013) recorded from the PFC of macaque monkeys while they performed a color or a motion discrimination task on the same stimuli. The authors found that the representation of the color and motion features, and of the choice the monkeys made were separable at the population level but intermixed at the single neuron level. Separation of function at the neural population level but not at the single neuron level in PFC has been shown in other studies as well (Sigala et al., 2008; Machens, 2010; Machens et al., 2010; Stokes et al., 2013). As discussed previously, analysis of response patterns in V1, V2, and V4 showed that neurons can have multiplexed but separable selectivities to color and form (McClurkin and Optican, 1996; McClurkin et al., 1996).

In light of the results on high level cognitive areas can we make any inferences on neural representations in early visual cortex? A prominent feature of non-linearly selective PFC cells is the complexity of their selectivity. Note that if the neural representation is high dimensional, a linear decoder can implement many input-output combinations; an attribute that is necessary for a population of neurons that perform complex tasks. As discussed earlier (Rigotti et al., 2013), neural populations that can perform complex tasks are suggestive of neurons with non-linearly mixed selectivities. In light of a number of studies showing that neurons in V1 can also show complex response properties previously attributed to higher order areas, we discuss in the next section the possibility that non-linearly mixed neurons are pervasive in the cortex.

## COMPLEX SELECTIVITY IN EARLY VISUAL CORTEX

Neurons in the early stages of visual processing respond to visual stimuli within a local region in space, the classical receptive field. However, the responses of neurons to stimuli within their classical receptive field do not fully encompass their properties. Stimuli outside the neurons’ classical receptive field do not elicit a response, but can modulate the response of the neurons to stimuli within their receptive field (Gilbert et al., 1996; Angelucci and Bressloff, 2006). An example of this modulation is surround suppression where, after a certain stimulus diameter, as the size of a stimulus centered within the receptive field of a neuron increases, the rate of firing of the neuron decreases (Hubel and Wiesel, 1965; Blakemore and Tobin, 1972; Nelson and Frost, 1978; Knierim and Van Essen, 1992; DeAngelis et al., 1994; Levitt and Lund, 1997; Adesnik et al., 2012). The properties of neurons in higher cortical areas are much more complex than the well-established classical and extra-classical receptive field properties of neurons in early visual cortex. Other studies, however, have indicated even more complex properties of cells in early visual cortex that may suggest that these cortical areas are more than just a relay to higher visual areas. As discussed in the previous section, complex selectivity of a population of neurons is indicative of non-linearly mixed selectivities at the single neuron level. Thus, complex selectivity in early visual cortex could suggest population of neurons that are responsive to several features.

Recent experiments confirm that activity in V1 can be driven or modulated by prior expectations. In an fMRI study, Kok et al. (2013) used a forward model to predict the direction of random dot motion patterns from activity in the early visual areas. Their results indicated that experimental priors can change the contents of the neural representation in early sensory cortex. Keller et al. (2012) showed that a subset of cells in the primary visual cortex of mice responded only when there was a mismatch between what the mouse was expecting to see and what it actually saw while it was running. Interestingly enough, the cells that showed the strongest responses could also encode the degree of mismatch between expectation and actual visual feedback. McManus et al. (2011) recorded from monkeys performing a contour detection task and found that V1 neurons were selective to complex forms and that this selectivity could be modulated by the monkeys’ expectation of the form.

Evidence from electrophysiological studies in monkeys has indicated that attention enhances the response of neurons with receptive fields that are within the focus of attention in all of the cortical areas along the ventral stream, including V1 (Moran and Desimone, 1985; Spitzer et al., 1988; Chelazzi et al., 1993; Luck et al., 1997; McAdams and Maunsell, 1999). Furthermore, it has been shown that V1 neurons have complex perceptual grouping properties previously assigned to higher areas. Lamme (1995) found that V1 neurons play a critical role in figure-ground segregation since they show response enhancement for stimuli presented in the figure compared to stimuli presented in the ground area. In a binocular disparity study, Sugita (1999) showed that some orientation selective neurons in V1 had a diminished response when bars were occluded by a patch, but restored their response when the patch had crossed disparity and thus appeared to be in front of the bars. The studies by Lamme (1995) and Sugita (1999) along with the study by Wachtler et al. (2003) discussed in the previous section suggest that the modulation of neurons depends on global context.

The studies, we discussed in this section, indicate that neurons in early visual cortex are highly context dependent. The proposition, that there exists a context dependent population of neurons which at the same time processes segregated features of the visual scene seems contradictory to us. Furthermore, the complex properties of cells discussed here suggest that the neural population code in early visual cortex is high dimensional. High dimensional codes arise from non-linearly mixed selectivities at the single neuron level. Non-linearly mixed selectivities at the single neuron level may also explain the often conflicting neurophysiological results in the early visual cortex on color and form processing. Population average response can hide some of the selectivities of a neural population. However, as the study by Wachtler et al. (2003) showed, consideration of the pattern of responses from a population of neurons can reveal the complex behavior of the neurons.

The hierarchical model of vision represents V1 cells as localized spatial filters that extract low level visual features to transfer this information to the higher levels. However, V1 cells’ complex perceptual grouping properties suggest strong influences from horizontal and feedback connections from higher visual areas. In the next sections we examine the feedforward and recurrent modes of processing and possible ways they can modulate color and form selectivity of early visual cortical areas.

## FEEDFORWARD vs. RECURRENT PROCESSING OF COLOR AND FORM

Thorpe et al. (1996) have shown that in a categorization task of complex natural images, ERP activity starts to differentiate for different visual targets approximately 150 ms after stimulus onset. Furthermore, a simple weighted summation of spike counts on a population of neurons in IT taken between 100–150 ms after an object is presented can decode object identity even with moderate changes in the object’s position, scale, illumination, pose and clutter (DiCarlo et al., 2012). These results along with other electrophysiological (Keysers et al., 2001) and psychophysical (Potter et al., 2014) studies suggest that processing in the visual system can be fast and use mainly if not exclusively feedforward circuits.

However, in the brain, we also find feedback connections from higher order areas to lower ones (Kennedy and Bullier, 1985; Shipp and Zeki, 1989; Felleman and Van Essen, 1991). Feedback connections enable the receptive field properties of neurons to change dynamically, in order to adapt to differences in behavioral state, contextual influences or expectations (Gilbert and Li, 2013). They can also contribute to the disambiguation of noisy scenes (DiCarlo et al., 2012).

As discussed earlier, there are cells as early as in V1 that code for both color and orientation (Leventhal et al., 1995; Friedman et al., 2003). A feedforward model similar to the one proposed by Hubel and Wiesel (1962) could explain this kind of tuning; a V1 cell has oriented, color selective regions in its receptive field because it receives synaptic input from center surround, color opponent LGN cells. This model is physiologically plausible. However, it is unlikely that all processing occurs this way.

Incremental grouping theory proposes a link between different perceptual grouping mechanisms and feedforward and recurrent processing (Roelfsema, 2006; Roelfsema and Houtkamp, 2011). It distinguishes perceptual grouping mediated by base and incremental grouping mechanisms. The base grouping mechanism groups feature conjunctions/objects that are coded by individual cells. The base grouping mechanism can code features of different complexities; it includes cells in V1 that code for both color and orientation and in medial temporal lobe that are selective to specific individuals (Kreiman et al., 2002; Quiroga et al., 2005). It is fast, feedforward and happens in parallel. However, it would lead to a combinatorial explosion if single neurons coded for every possible combination of objects/features. The incremental grouping mechanism is used for objects/features that are not coded by single specialized neurons. It increases the response of a population of neurons that encode the features to be grouped via feedback and horizontal connections. This process is slow since the spread of neural enhancement resulting in perceptual grouping happens gradually. There has been neurophysiological evidence in support of a distinction between base and incremental processing (Roelfsema et al., 1998, 2004; Pooresmaeili et al., 2010). A recent study showed that in macaque, V1 integration spreads out in an approximately 300 ms period from the focus of attention, following perceptual grouping criteria (Wannig et al., 2011). Thus depending on the situation at hand, visual processing can operate in two modes: the feedforward one which is specific, strong and fast and the feedback one which is diffuse, weak and slow.

In incremental grouping, color and orientation are jointly coded during feedforward processing. Recurrent processing, however, can possibly change dynamically the weights of color and orientation selectivity in early visual areas. Neurons as early as in V1 can slowly enhance their response via recurrent influences to signal the association of features to an object (Roelfsema et al., 1998). This may depend on contextual influences, including task or prior expectations.

Predictive coding theory also suggests a mode of visual processing different from the segregated one. The theory states that perceptual, cognitive and action-oriented processing follow a single general strategy, which uses top-down predictions to minimize prediction errors (Clark, 2012). This approach suggests that neuronal selectivity to a feature is not an intrinsic property but the result of interactions across levels of a processing hierarchy (Friston, 2003). Sensory neurons, rather than features *per se*, encode an error signal, i.e., they feed forward to hierarchically higher areas the discrepancy between the actual input and the top-down expectation (Egner et al., 2010). According to the predictive coding model, predictions are relayed via feedback connections, whereas prediction errors are conveyed via feedforward connections (Rao and Ballard, 1999). Hosoya et al. (2005) showed that retinal ganglion cells’ spatio-temporal receptive fields change dynamically with the visual scene; this result is in line with the view that the raw signal, carried by the receptors, is transformed as early as in the retina by the first interneurons which encode deviations from predicted temporal and spatial structures (Srinivasan et al., 1982). Recent fMRI studies have also shown evidence in support of predictive coding in the visual cortex (Summerfield et al., 2008; Kok et al., 2012). For instance, Kok et al. (2012) found that the amplitude of the fMRI signal in early visual cortex was smaller when the stimulus was expected; typically when we see something that we expect the prediction error encoded in the brain is smaller compared to when we see something unexpected. This mode of processing, however, appears to be at odds with several electrophysiological studies (see Koch and Poggio, 1999 for a commentary).

In the predictive coding framework, context determines whether sensory neurons perform segregated or integrated processing. For example, if the color of some colorful shape is unexpected the visual system generates the prediction error related to the color only. As a result the neural response will indicate color selectivity which is segregated from form. However, if both the color and shape are unexpected, the prediction error will have information about both features, and the neural response will reflect integrated processing.

## CONCLUSION

Early studies on visual processing indicated that different regions in the brain show biases in their selectivity for color and form. These results suggest that color and form are processed by distinct modules in the visual cortex. All of these studies assumed in their analyses that each neuron is an independent computational unit; thus weak selectivities at the single neuron level were disregarded. Meanwhile, these results were found to be at odds with some psychophysical and electrophysiological observations which suggested integrated processing of color and form in early visual cortex.

Studies on higher cortical areas have shown that visual representations and complex task conditions are represented by the distributed activity of a population of neurons. Here selectivities to a feature that appear weak at the single neuron level may encode that same feature robustly at the population level. Populations of neurons that perform complex tasks in PFC were shown to have non-linearly mixed selectivities at the single neuron level.

Recent studies have showed that early visual areas are not just passive relays of local information, but rather complex processing stages that incorporate global context and prior information. This behavior arises from the flow of information from horizontal and feedback connections that can dynamically adapt the selectivities of single neurons to the situation at hand. The increasing evidence that the early visual areas show this kind of complex selectivity suggests that population codes operate in a high dimensional space; this property makes it likely that single neurons have non-linearly mixed selectivities. Examining these selectivities at the single neuron level can be misleading. Based on the above evidence, we argue that color and form features not only are continuously interacting in our visual experience, but are also integrated rather than segregated in the visual cortex.

## Conflict of Interest Statement

The authors declare that the research was conducted in the absence of any commercial or financial relationships that could be construed as a potential conflict of interest.
